# The prognostic effect of PTEN expression status in colorectal cancer development and evaluation of factors affecting it: miR-21 and promoter methylation

**DOI:** 10.1186/s12929-016-0228-5

**Published:** 2016-01-19

**Authors:** Yaghoub Yazdani, Touraj Farazmandfar, Hossein Azadeh, Zeinab Zekavatian

**Affiliations:** Infectious Diseases Research Center and Laboratory Science Research Center, Golestan University of Medical Sciences, Gorgan, Iran; Student Research Committee, Golestan University of Medical Sciences, P.O. Box: 4934174611, Gorgan, Iran; Department of Internal Medicine, Mazandaran University of Medical sciences, Sari, Iran; Department of Genetics, Tehran Medical Sciences branch, Islamic Azad University, Tehran, Iran

**Keywords:** Colorectal cancer, PTEN expression, miR-21, PTEN promoter methylation

## Abstract

**Background:**

PTEN is a tumor suppressor gene which is involved in cellular proliferation, differentiation, and apoptosis. Loss or down-regulation of PTEN plays an important role in human cancers development. In this study, we investigated the effect of miR-21 and promoter methylation on the PTEN expression status in CRC tissues and analyzed association of the PTEN expression status with clinicopathological features in patients with CRC.

**Results:**

The PTEN expression was positively detected in 67.2 % CRC tissues and all adjacent non-cancerous samples. PTEN mRNA level was negatively correlated with miR-21 level (r = −0.595, *P* < 0.001). PTEN expression was also correlated directly with the PTEN mRNA level (r = 0.583, *P* < 0.001) and conversely with miR-21 level (r = −0.632, *P* < 0.001). PTEN Promoter methylation was significantly associated with PTEN expression status (*p* = 0.013). PTEN expression was negatively associated with tumor size (*p* = 0.007) and advanced tumor stage (*P* = 0.011). Multivariate analysis indicated that tumor stage, tumor differentiation and PTEN expression status were independent prognostic factors for overall carcinoma in CRC patients (*P* < 0.05). The Kaplan-Meier curve indicated a negative correlation between PTEN expression levels and survival of CRC patients (*P* = 0.013).

**Conclusions:**

This study suggests a high frequency of miR-21 overexpression and aberrant promoter methylation in down-regulation of PTEN expression in colorectal carcinoma. Loss of PTEN may be a prognostic factor for patients with CRC.

## Background

Colorectal cancer (CRC) is the third commonly diagnosed cancer worldwide, with approximately 1.3 million new cases and half million deaths annually [1]. The development of CRC from normal endothelium to advanced carcinomas involves a multiple process with accumulation of genetics and epigenetics changes, leading to a high activity of oncogenes and low activity or dysfunction of tumor suppressor genes [2]. To date, despite great effort to clarifying the molecular mechanisms in CRC, the molecular pathogenesis of CRC remains unclear.

PTEN (phosphatase and tensin homolog deletion on chromosome 10) is a tumor suppressor protein with the phosphatase activity and acts as a negative regulator in the PI3K/AKT pathways. This pathway control several processes related to cell metabolism, proliferation and survival. PTEN plays an essential role in the silencing of signal transduction from several membrane growth factor receptors (Her1, Her2 and IGFR) through the PI3K/AKT signaling cascade [3, 4]. Apart from phosphatase activity, PTEN forms a nuclear complex with p53 protein, a tumor suppressor protein. This complex inhibits p53 decomposition and increases its transcriptional activity [5, 6]. Nuclear PTEN induce arrest in the G0-G1 phase of cell cycle by down-regulation of cyclin D1 and ERK/MAPK pathway [7]. In many tumor types, genetic alterations of *PTEN* gene enhance tumorigenesis and may determine aggressive clinicopathological behavior of a tumor [8–11]. The role of PTEN in CRC was postulated earlier as one of the factors of PTEN hamartoma tumor syndrome that the estimated lifetime risk of CRC in these patients is 9 % [9, 11]. Previous studies indicate that *PTEN* gene mutations in sporadic CRC are rare [12–14] and other mechanisms might be involved in inactivation of PTEN, such as promoter hypermethylation and microRNAs [15, 16].

MicroRNAs are a class of short non-coding RNAs with 18–25 nucleotides in length. Those negatively regulate gene expression by complementary binding to the 3′-untranslated region of target mRNAs; this causes translation inhibition or mRNA degradation [17]. Many Studies have demonstrated the important role of microRNAs in almost all cellular processes including proliferation, metabolism, differentiation, apoptosis and the immune response [18–20]. MicroRNAs have been demonstrated to play a significant role in the multi-step process of carcinogenesis [21, 22]. Recent reports show that miR-21 is consistently overexpressed in many types of tumors. PTEN mRNA has been known as one of miR-21 targets that significantly associated with several malignancies in human [23–25]. However, the expression levels of miR-21 and PTEN mRNA have not been sufficiently studied in CRC.

DNA methylation is an important mechanism in epigenetic control, which has been involve in the development of many of cancers [26]. Hyper-methylation of some tumor suppressor genes has been related to the initiation and development of various human cancers [27, 28]. Hypermethylation of *PTEN* gene contributes to CRC development is not yet clarify and needs more studies.

In this study, we assayed impact of miR-21 and promoter methylation on the PTEN expression status in CRC tissues and analyzed correlation of the PTEN expression with clinicopathological features in CRC patients.

## Methods

### Subjects

One hundred and twenty-five samples of Formalin-fixed paraffin-embedded (FFPE) colorectal carcinomas and adjacent non-cancerous tissues were collected from the sporadic CRC patients who had surgery between March 2005 and October 2011. None of the patients were treated by chemotherapy or radiotherapy before surgery. Clinicopathological features of patients were obtained from medical records. The survival time was considerate from the diagnosis date to the last follow-up date. This study was approved by the Clinical Research Ethics Committee in Golestan University of Medical Science.

### Immunohistochemistry (IHC)

PTEN IHC was performed on 5 μm unstained micro-dissected tissue blocks using a mouse monoclonal antibody (6H2.1, Dako, California, USA) (1:100 dilutions). After deparaffinization, antigen was retrieved on sections (100 °C, pH = 9.0, 25 min). The sections were then submerged in anti-PTEN antibody (35 °C, 15 min). Subsequently, they were submerged in hydrogen peroxide (35 °C, 5 min) for deactivation of the endogenous peroxidase. Following these steps, the sections were covered with secondary anti-mouse immunoglobulin (35 °C, 8 min). Diaminobenzidine was applied as a chromogen and sections were counterstained using hematoxylin. The stained slides were examined using light microscopy (Olympus BX41, Richmond Hill, Canada). The percentage of PTEN immunostaining tumor cells was identified and a semiquantitative scoring system was applied to evaluate the staining results (0, < 5; 1+, 5–25; 2+, 25–50; and 3+, > 50 %).

### Primer design

The amplification primers for specific-recognizing of PTEN complementary DNA (cDNA) and Hypoxanthine-guanine phosphoribosyltransferase (HPRT) cDNA were designed by Gene Runner software (version 3.05; Hastings, USA). Methylation-Specific PCR (MSP) primers of PTEN promoter were designed as described by Zysman et al. [29]. All primers were reviewed in NCBI and BLAST websites (Table 1).

**Table 1:** The used primers in this study

Amplified factor	primers 5′ to 3′		Product size (bp)	AT (°C)	Genbank accession number
miR-21	S	TGTCGGGTAGCTTATCAGAC	~90	55	NR_029493
	AS	Adapter primer in kit			
U6-snRNA	S	GCTTCGGCAGCACATATAC	~120	55	NR_004394
	AS	Adapter primer in kit			
PTEN	S	ACCAGAGACAAAAAGGGAGTA	173	56	NM_000314
	AS	ACCACAAACTGAGGATTGCA			
HPRT	S	TGGACTAATTATGGACAGGACT	219	56	NM_000194
	AS	CCTGTTGACTGGTCATTACAAT			
PTEN Promoter					
Unmethylated (−300)^a^	S-U	TGGGTTTTGGAGGTTGTTGGT	173	55	NG_007466.2
	AS-U	ACTTAACTCTAAACCACAACCA			
Methylated (−298)^a^	S-M	GGTTTCGGAGGTCGTCGGC	155	57	
	AS-M	CAACCGAATAATAACTACTACGACG			

*S* sense, *AS* antisense, *U* unmethylated, *M* methylated, *AT* annealing temperature

^a^Distance of the 5′ nucleotide of the sense primer from the transcription start site of *PTEN* gene

### Quantitative reverse transcriptase Polymerase Chain Reaction (QRT-PCR)

Total RNA was extracted from micro-dissected FFPE weighing 50 mg by PureLink FFPE RNA Isolation Kit (Invitrogen, Carlsbad, USA) and modified to first strand cDNA using miScript II RT Kit (Qiagen, Hilden, Germany) according to the manufacturer’s protocol. For analysis of PTEN mRNA levels, QRT-PCR was performed using primers set and by Maxima SYBR Green/ROX qPCR Master Mix (Fermentas, Sankt Leon-Rot, Germany). MiR-21 was also quantified using the forward primer and miScript SYBR Green PCR Kit (Qiagen, Hilden, Germany) according to the manufacturer’s protocol. Each sample was tested in triplicate, in 7500 Real-Time PCR system (Applied Biosystem, Foster City, USA) and was normalized to endogenous control. The expression level was calculated using average of the 2^-ΔCt^ (ΔCt = Ct of control gene - Ct of target gene) [30].

### Promoter methylation assay

Genomic DNA (1 μg) of micro-dissected tumor tissues was extracted by the MagMA FFPE DNA Isolation Kit (Invitrogen, Carlsbad, USA) according to the manufacturer’s protocol. Bisulfite modification was performed by EpiTect Fast Bisulfite Conversion Kit (Qiagen, Hilden, Germany) according to the manufacturer’s protocol. MSP was performed using 100 ng of bisulfite-modified DNA, primers set and Taq DNA Polymerase Master Mix (Ampliqon, Copenhagen, Denmark) in a final volume of 25 μl in thermal cycler instrument (Eppendorf, Hamburg, Germany). MSP were analytically validated using standard methylated DNA as positive control (Chemicon, Temecula, USA) and primary keratinocyte DNA as negative controls. The amplification products were electrophoresed on a 2.5 % agarose gel, stained in sybr green, and visualized by UV transilluminator (Uvitec, Cambridge, UK). The MSP results were reported as methylated samples (a band 173 bp) and unmethylated samples (a band 155 bp). MSP results of PTEN promoter were also confirmed by bisulfite sequencing.

### Statistical analysis

The correlation of PTEN expression status to PTEN mRNA level and miR-21 levels was tested by Spearman correlation analysis. The association between PTEN expression status and Clinicopathological features were analyzed by ANOVA and Fisher’s exact tests. The survival curves were estimated by the Kaplan-Meier method and the log rank test was applied to compare the differences between curves. Multivariate analysis was performed by the Cox regression model. Data were analyzed using SPSS software version 17.0 (SPSS Inc. Chicago, USA). A p-value of less than 0.05 was considered statistically significant.

## Results

### PTEN expression and factors affecting it in CRC

**Fig. 1:**
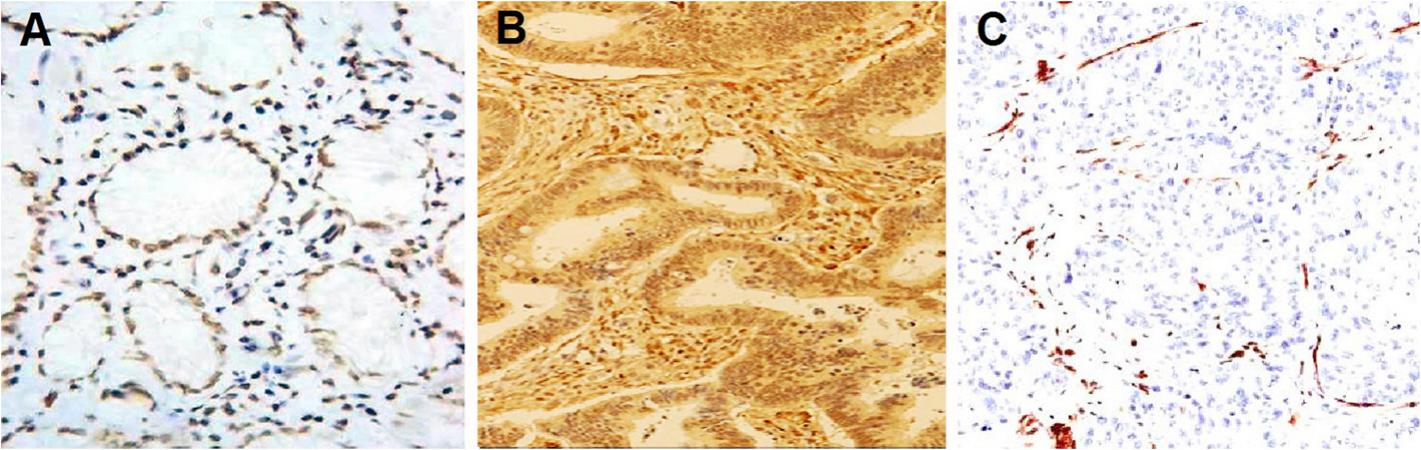
Representative IHC staining of colorectal samples. **a** PTEN was positively expressed in adjacent normal tissue (**b**) and colorectal carcinomas. **c** Negative expression for PTEN was observed in some CRC tissues samples (x200)

Positive PTEN staining was detected in the nucleus of corresponding normal mucosa (Fig. 1a) or carcinoma cells (Fig. 1b). In some cancerous samples, no PTEN expression was observed (Fig. 1c). PTEN expression was positively (1+, 2+ and 3+) detected in 67.2 (84 of 125) of CRC tissues and 100 % of adjacent non-cancerous samples. PTEN expression in CRC tissues was statistically lower than the non-cancerous mucosa (*P* < 0.001) (Table 2).

**Table 2:** PTEN expressions in CRC tissues and matched non-cancerous tissues

Tissue type	N.	PTEN expression					*P*-value
		0	1+	2+	3+	PR (%)	
Non-cancerous	125	0	18	59	48	100	<0.001
Cancerous	125	41	34	37	13	67.2	

*PR* positive rate

**Fig. 2:**
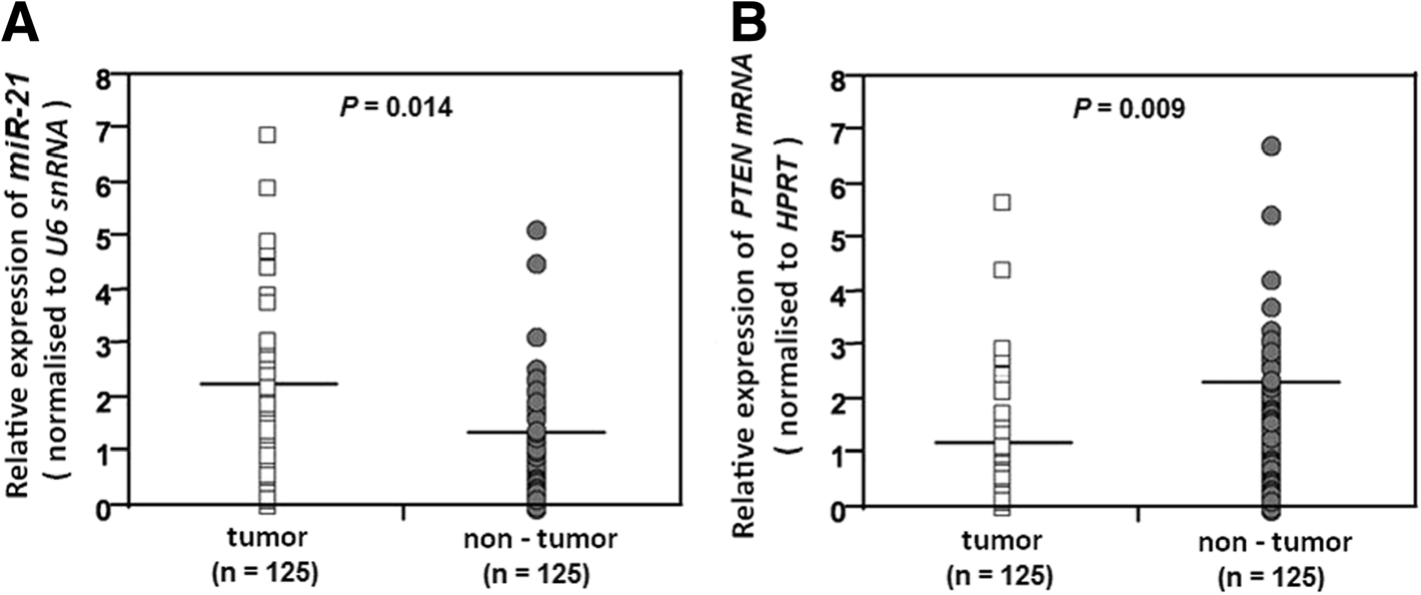
QRT-PCR analysis of miR-21 and PTEN mRNA in CRC patients. **a** The level of miR-21 in tumor tissues was significantly higher than non-tumor tissues. **b** Level of PTEN mRNA in tumor tissues was significantly lower than non-tumor tissues. Horizontal lines indicate the mean values. Differences between groups were analyzed using the Student’s t-test

**Fig. 3:**
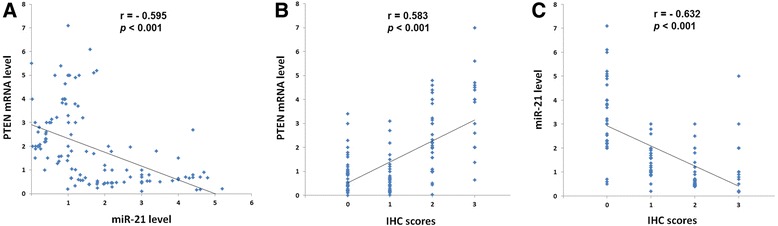
Scatter plots for three correlation outcomes in CRC tissue samples. **a** Scatter plot for correlation analysis between PTEN mRNA level and miR-21 level, **b** PTEN mRNA level and IHC scores, **c** and miR-21 level and IHC scores

The results indicated that the average of miR-21 level (Mean ± SD, 2.113 ± 0.652) in cancerous tissues was significantly high compared with normal tissues (1.211 ± 0.512) (*p* = 0.014) (Fig. 2a). In contrast, the level of PTEN mRNA was significantly down-regulated in tumor tissues (1.278 ± 0.712) compared with normal tissues (2.291 ± 0.935) (*p* = 0.009) (Fig. 2b). The correlation coefficient test indicated that PTEN mRNA level was negatively correlated with miR-21 level (r = −0.595, *P* < 0.001) (Fig. 3a). PTEN expression was also correlated directly with the PTEN mRNA level (r = 0.583, *P* < 0.001) (Fig. 3b) and conversely with miR-21 level (r = −0.632, *P* < 0.001) (Fig. 3c).

**Fig. 4:**
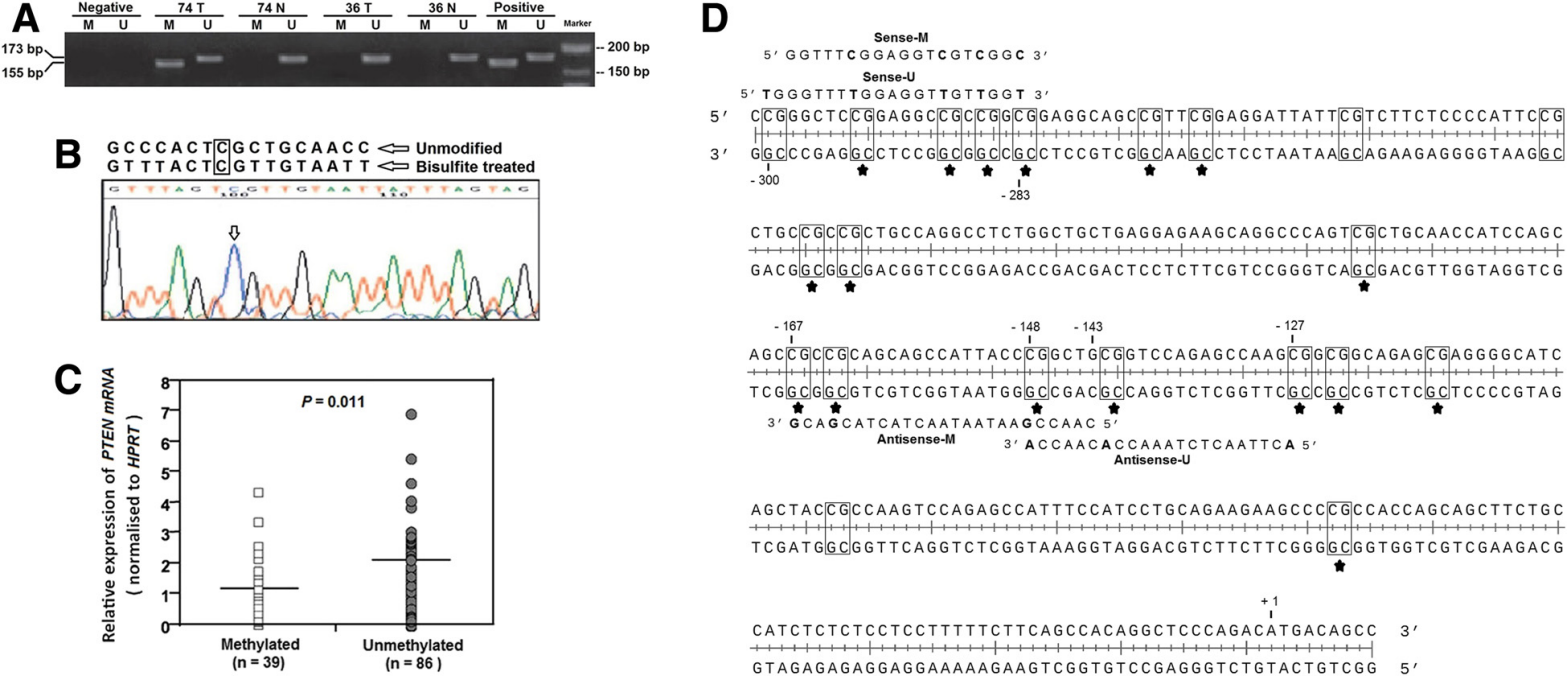
Representative results of MSP analysis of the PTEN gene in CRC patients. **a** The Methylation status was determined based on Product size-band; 176 bp for unmethylated and 155 bp for methylated. Universal methylated and unmethylated DNA was used as a positive control. Water was used as a negative control. N, normal tissue; T, tumor tissues; M, methylated; U, unmethylated. **b** Bisulfite sequencing of the PTEN promoter was performed using primers of methylation analysis on the methylated samples. Bisulfite sequencing results indicated that the sequence shown in the electropherograms matched the PTEN gene at the one critical nucleotide positions (marked with arrows and box). **c** The level of PTEN mRNA in methylated samples was significantly lower than unmethylated samples. Horizontal lines indicate the mean values. Differences between groups were analyzed using the Student’s t-test. **d** Part of the PTEN promoter region. +1 indicates the translation start site and the location of the primer set is marked using number. Differences between unmethylated and methylated sequences on primers are shown in bold. The CpG sites are in box and common methylated sites in all methylated samples are marked by asterisk

MSP analysis of CRC tissues using PTEN promoter specific primers showed that 39 samples (31.2 %) have been methylated (Fig. 4a). As Table 3 shows, 85 % (33 of 39) of the methylated CRC samples showed loss or reduced PTEN protein staining (0 and 1+). Comparison of methylated and unmethylated groups based on the IHC scores, shows that the frequency of PTEN Promoter methylation has decreased in groups with the increased IHC staining score. (*p* = 0.013). We studied normal tissues for investigation of the possibility of PTEN promoter methylation, and none of those showed aberrant methylation (Fig. 4a). The MSP data were confirmed by bisulfite sequencing on some of the methylated sporadic CRC samples. These data clearly showed that specific sequence belonged to the PTEN gene promoter and not the pseudogene (Fig. 4b). Moreover, level of PTEN mRNA was significantly high in unmethylated samples (2.452 ± 1.536) compared with methylated samples (1.213 ± 0.671) (*p* = 0.011) (Fig. 4c).

**Table 3:** Relationship between PTEN expression and clinicopathological data in CRC patients

Clinicopathological features	N.	PTEN protein expression					*P*-value
		0	1+	2+	3+	PR (%)	
Total cases (cancerous)	125	41	34	37	13	67.2	
Age							0.943
< 50	18	6	4	6	2	66.7	
≥ 50	107	35	30	31	11	67.2	
Sex							0.251
Female	55	15	16	18	6	72.7	
Male	70	26	18	19	7	62.8	
Tumor size (cm)							0.007
≤ 5	79	19	23	27	10	76	
> 5	46	22	11	10	3	52.2	
TNM Stage							0.011
I & II	25	3	6	12	4	88	
III & IV	100	38	28	25	9	62	
Tumor Differentiation							0.534
High	65	19	17	18	11	70.8	
Moderate	49	17	14	16	2	65.3	
Low	11	5	3	3	0	54.5	
PTEN Promoter methylation							0.013
Methylated	39	18	15	4	2	53.8	
Unmethylated	86	23	19	33	11	73.2	

*PR* positive rate, *TNM* Tumor, *Node* Metastases staging system

### PTEN expression status and clinicopathological features

**Fig. 5:**
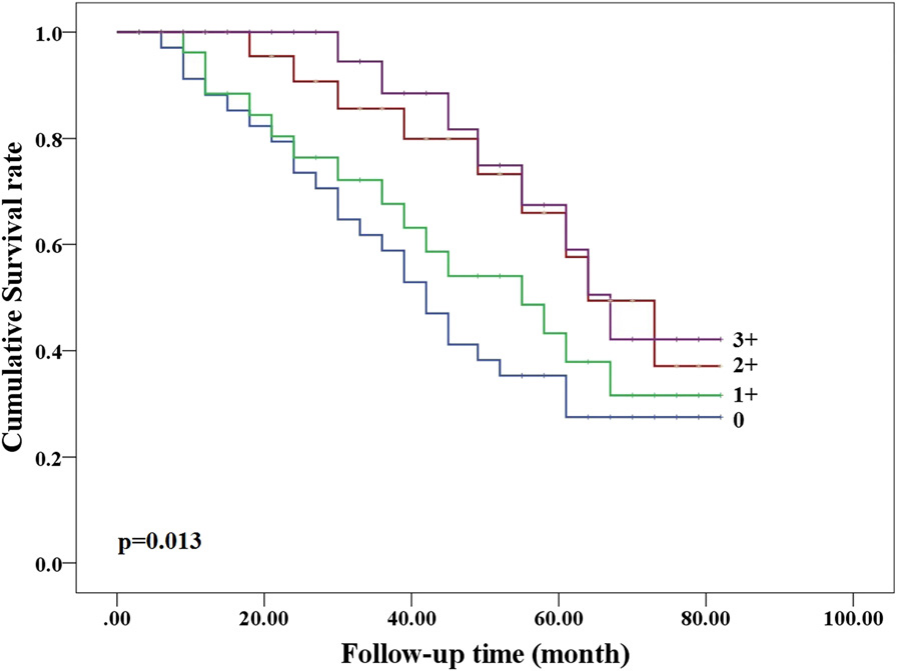
Kaplan-Meier curves for overall survival in patients with CRC. The overall survival has increased in accordance with the increased IHC score

Comparison of clinicopathological groups based on the IHC scores have been shown in Table 3, positive PTEN expression was associated negatively with tumor size (*p* = 0.007) and positively with advanced tumor stage (*P* = 0.011), but no association was observed with age, gender and tumor differentiation (P > 0.05) in CRC patients. Multivariate analysis using Cox’s proportional method indicated that tumor size, tumor stage and PTEN expression status were independent prognostic factors in overall CRC (*P* < 0.05) (Table 4). Follow-up information was available on 101 CRC patients for 40 ± 22 (3 to 80) months. Kaplan-Meier curve indicated a negative correlation between PTEN expression levels and survival of CRC patients. It shows that the overall survival has increased in accordance with the increased IHC score. (*P* = 0.013) (Fig. 5).

**Table 4:** The multivariate analysis of clinicopathological variables for overall survival in CRC patients

Clinicopathological variables	Relative risk (95 % CI)	*P*-value
Age (≥50)	0.980 (0.70 - 1.12)	0.352
Sex	1.26 (0.81 - 1.78)	0.237
Tumor size (>5 cm)	2.11 (1.01 - 3.97)	0.010
Stage ( III & IV)	1.59 (0.73 - 2.89)	0.013
Differentiation (low)	1.51 (1.03 - 2.44)	0.094
PTEN expression	2.23 (1.06 - 4.68)	0.008

*CI* Confidence Interval

## Discussion

*PTEN* is a tumor-suppressing gene which is involved in cellular metabolism, proliferation, differentiation, and apoptosis. Loss or down-regulation of PTEN plays an important role in development and progression of malignancies [3–6]. The different mechanisms may be involved in the regulation of PTEN expression including mutations, promoter methylation, microRNAs, posttranslational regulation, and the PTEN protein stability [3, 10, 24]. Among these factors, Down-regulation of *PTEN* gene by promoter methylation and microRNA targeting has been reported in many types of human cancers [21–24, 27, 28]. Recent studies about *PTEN* gene dosage on both humans and mice indicated that even partial loss of function is sufficient for development of some cancer types [31], suggests that the evaluation of minor changes in PTEN expression is crucially important. In the present study, we measured the expression of PTEN mRNA and protein in CRC tissues and analyzed the effect of miR-21 level and aberrant promoter methylation on reduction or loss of PTEN expression. In this study, IHC results shows 67.2 of CRC samples were PTEN-positive and 32.8 % were negative (Table 2), that is closed to previous studies results [32, 33]. MiR-21 is one of the most important microRNAs in human cancer and is known as a potential oncogene. MiR-21 is up-regulated in various carcinoma types and has been correlated with poor therapeutic outcome and poor survival [34–36]. In the present study, the level of miR-21 was significantly higher in cancerous tissues than normal tissues (Fig. 2a). Correlation analysis of IHC results also shown, MiR-21 level was negatively correlated with PTEN expression (Fig. 3). In addition, miR-21 was over-expressed in 75.5 % cancerous tissues with reduced or lost PTEN protein. These findings confirmed role of miR-21 in regulation of PTEN expression in CRC. These results are consistent with other studies [34–39] suggesting that the high expression of miR-21 might be an early diagnosis marker in CRC. Epigenetic silencing of *PTEN* gene by promoter methylation was initially proposed in prostate cancer cell lines and identified as an effective mechanism in melanoma development [40]. The genomic sequence of PTEN promoter is very identical to 841 bp in a highly conserved region on *PTEN* pseudogene. As a consequence, the extreme caution needs to select primer set when analyzing the PTEN promoter methylation [29]. Following these recommendation, in this study, we reported a 31.2 % frequency for PTEN promoter methylation in CRC tissues. Moreover, we found that 85 % of the methylated CRC samples showed reduced or lost PTEN protein (Table 3), suggests high frequency of PTEN aberrant promoter methylation in colorectal carcinoma. These findings along with other results [15], suggest that the PTEN promoter methylation might be cancer specific and plays a role in the development of these neoplasms. Low expression PTEN mRNA in tumor and methylated samples compared with non-tumor and unmethylated samples (Fig. 2) demonstrated that PTEN expression status is a critical modulator in CRC development. We found that negative PTEN expression was statically associated with tumor size and advanced TNM stages in patients with colorectal carcinoma (Table 3), similar to a Waniczek et al. and Chow et al. studies [4, 41] but in contrast with Goel et al. study [15]. Multivariate analysis also showed for the first time that tumor size is an independent prognostic factor for overall carcinoma in CRC patients with low or lost PTEN expression (Table 4). In agreement with Jang et al. study [42], Survival analysis of present study indicated that the patients with loss of PTEN expression showed poorer survival than the patients with normal expression. These results were suggested that down-regulated expression of the PTEN protein probably contributed to growth, invasion, and metastasis of colorectal carcinoma and could be considered as a good marker to indicate the aggressive behaviors and poor prognosis of colorectal carcinomas. The results of this study and other similar studies indicated that the PTEN loss is a key factor in tumor development and its expression regulation may be a good target for developing drugs to prevent cancer progression in the feature.

## Conclusions

This study suggests a high frequency of miR-21 overexpression and aberrant promoter methylation in down-regulation of PTEN expression in colorectal carcinoma. Loss of PTEN may be a prognostic factor for patients with CRC. Analysis of PTEN expression profile may be a useful test for prognosis of CRC and reduces the cost of unnecessary use of antiviral drugs against growth factor receptors.
